# From sites to structure to serology: a roadmap for structure-aware molecular evolution of antigenically evolving viruses

**DOI:** 10.1128/jvi.01687-25

**Published:** 2026-07-16

**Authors:** Sanni Översti, Spyros Lytras, Shusuke Kawakubo, Jumpei Ito, Mahan Ghafari

**Affiliations:** 1 Department of Biology, University of Oxford 6396, Oxford, United Kingdom; 2 Antigen Evolution & Design Lab, Department of Structural Biology and Chemistry, Institut Pasteur 27058, Paris, France; 3 Division of Systems Virology, Department of Microbiology and Immunology, The Institute of Medical Science, The University of Tokyo, Tokyo, Japan; 4 Laboratory of Virus Informatics, Department of Biological Informatics, Bioinformatics Center, Research Institute for Microbial Diseases, The University of Osaka, Suita, Japan; 5 Center for Advanced Modalities and Drug Delivery Systems, The University of Osaka, Suita, Japan; 6 Pandemic Sciences Institute, Nuffield Department of Medicine, University of Oxford 685411, Oxford, United Kingdom; 7 Lincoln College, University of Oxford 6396, Oxford, United Kingdom; Universiteit Gent, Merelbeke, Belgium

**Keywords:** molecular evolution, antigenic evolution, antigenic cartography, protein structure-function, machine learning

## Abstract

The genomic deluge has pushed viral molecular evolution into a site-resolved era. For antigenically evolving viruses such as influenza and severe acute respiratory syndrome coronavirus 2 (SARS-CoV-2), dense genomic sampling now supports mutation-annotated phylogenies and per-site estimates of mutation and substitution processes. These data highlight strong effects of sequence context, genomic region, RNA structure, and protein-level constraints that are blurred by classic uniform substitution models. In parallel, accurate structure prediction and emerging structure-aware phylogenetic and machine-learning approaches provide practical ways to map mutations onto three-dimensional constraints, identify structurally plausible escape routes, and interpret evolutionary rate variation through solvent exposure, packing, stability, glycosylation, receptor-binding interfaces, and epitope geometry. Finally, antigenic cartography translates some forms of genetic change into an epidemiologically meaningful phenotype—antigenic distance—while predictive modeling increasingly enables sequence-to-antigenicity inference for variants that have not yet been tested experimentally. Here, we outline a practical framework linking sites, structure, and serology for viruses in which antigenic evolution is a major component of immune escape and lineage turnover; highlight why genetic and antigenic “clocks” can diverge; and discuss how integrating genomic surveillance data, phylogenetics, structural analysis, and predictive modeling could support more prospective variant assessment and improved vaccine and therapeutic design.

## INTRODUCTION

Advances in virus genomics have made it increasingly possible to track evolutionary changes at the population level over the timescales of outbreaks and epidemics (1). Before the current era of large-scale genomic surveillance, our inferences relied on sparse, heterogeneous datasets—dozens to hundreds of sequences, often from partial genomic regions, collected over many years. Such data sets were sufficient to estimate broad patterns of molecular evolution, identify major lineages and population structure, and detect signatures of selection, but they rarely allowed us to reconstruct when particular mutations arose, how they combined within genetic backgrounds, which changes persisted and contributed to lineage growth, or how they translated into measurable viral phenotypes.

High-throughput sequencing has changed that constraint for a growing set of viruses by turning evolution from something we infer retrospectively into something we can increasingly watch unfold. The most dramatic example is severe acute respiratory syndrome coronavirus 2 (SARS-CoV-2), for which millions of genomes—sampled across countries, time, and transmission settings—enabled pandemic-scale phylogenies, catalogs of recurrent changes, and empirical estimates of how mutation and selection vary across sites, genes, and lineages (2–4). That scale made it possible to ask questions that were previously out of reach, including which substitutions repeatedly arise in independent genetic backgrounds, which combinations of changes assemble on the same branches, and how quickly lineages with particular mutational profiles expand or disappear over the course of an outbreak. For other pathogens under sustained surveillance, particularly influenza viruses, the global number of available sequences remains far smaller than for SARS-CoV-2, and sampling is more heterogeneous, but is increasingly sufficient to reconstruct mutation-annotated trees across seasons and regions and to relate genetic turnover to antigenic change and epidemic dynamics (5–9). The shared payoff is resolution: dense, time-resolved sampling makes it possible to ask not only *what* changed, but *how* and *why* particular constellations of mutations emerge, spread, and persist.

A central question is how these patterns of genetic change alter viral fitness over time. Fitness is an aggregate outcome of multiple phenotypic components, including cell entry, replication efficiency, transmission, and immune escape, whose relative importance varies over time and across viruses, hosts, and epidemiological settings. For the class of endemic respiratory viruses that motivate much of this review—notably seasonal influenza A(H3N2) and, increasingly, SARS-CoV-2—antibody-mediated immune escape is often a key axis along which otherwise comparable lineages are differentiated, particularly in immune-experienced populations (7, 10).

For these viruses, serological assays provide a practical window onto one important immunological phenotype: antigenicity. Hemagglutination inhibition (HI) and neutralization assays—summarized through antigenic cartography (11)—offer an operational measure of how far a new variant has moved in “antigenic space” relative to strains that shaped existing immunity. This phenotype has long informed vaccine updates for influenza and, more recently, variant risk assessments for SARS-CoV-2. Genomics has often followed the serological signal: once an antigenically divergent variant is recognized, we ask which genetic changes distinguish it from previously circulating viruses (12).

Biologically, however, the causal chain runs in the opposite direction. Mutations at specific sites alter protein structure and surface chemistry, which, in turn, change phenotypes such as antibody binding, glycan shielding, receptor affinity, and protein stability, and these phenotypic effects can ultimately shape lineage success in host populations (10, 13). In influenza, substitutions in hemagglutinin (HA) and neuraminidase (NA) glycoproteins can remodel antibody epitopes, add or remove glycan shields, and alter receptor binding or stability (13–15). Although seasonal influenza vaccine effectiveness is primarily driven by the antigenic characteristics of HA, emerging evidence of asynchronous antigenic drift between HA and NA highlights the potential to enhance vaccine effectiveness by standardizing NA inclusion in seasonal vaccine design (16). Alternatively, the antigenic thrift theory—proposing that influenza evolution is shaped by immune responses targeting epitopes of limited variability (ELVs) within the HA head region—may potentially offer a route toward universal vaccines (17–19). However, further research is needed to evaluate the applicability of both approaches in future influenza vaccine design.

In SARS-CoV-2, changes concentrated in the spike glycoprotein—especially the receptor-binding domain (RBD) and N-terminal domain (NTD)—can reshape dominant antibody footprints and shift the balance between immune escape, angiotensin-converting enzyme 2 (ACE2) affinity, and trimer stability (10, 20, 21). Yet reading this chain from data, and forecasting it prospectively, remains difficult for several related reasons. First, only a minority of mutations have large, interpretable effects on the phenotypes we care most about, whereas many are removed by purifying selection or persist only because they hitchhike in successful backgrounds (22). Second, epistasis is pervasive: the same mutation can have different antigenic or functional consequences depending on genetic background, and successful variants often carry constellations that resolve trade-offs (23, 24). Third, genetic and antigenic changes are not simply coupled: sequence divergence may accumulate in an approximately clock-like fashion, while antigenic evolution can be more irregular and episodic (7). Finally, the fitness consequences of mutations depend on a changing host immune landscape, so the same mutation or antigenic shift need not have the same epidemiological significance across time or populations.

Closing this gap motivates integrating three views that have too often been treated separately: *sites*, which capture what changes arise, where they occur, and how often; *structure*, which captures what those changes do mechanistically and what constraints they face; and *serology*, which captures the extent to which existing antibody-mediated immunity still recognizes circulating variants.

### When does the sites–structure–serology roadmap apply?

This framework is not intended to apply uniformly to all viruses. Its strongest domain of application is viruses for which antigenic change is both measurable and epidemiologically consequential, particularly viruses circulating in immune-experienced host populations where antibody escape contributes to lineage turnover. Seasonal influenza A(H3N2) and SARS-CoV-2 are therefore useful motivating examples because they combine dense genomic surveillance, experimentally tractable surface glycoproteins, available structural data, and increasingly rich serological data sets.

Other viruses occupy different evolutionary regimes. In stability-dominated systems such as measles virus, antigenic drift is strongly constrained, and long-standing vaccine strains remain protective against contemporary circulating viruses (25, 26). Poliovirus provides a more nuanced example: antigenic changes can occur, particularly during prolonged circulation or vaccine-derived evolution, but population-level persistence is not generally driven by continuous antigenic drift in the same way as for seasonal influenza A(H3N2) (27). Conversely, chronic infections such as HIV and HCV generate substantial within-host diversity under immune and therapeutic pressures (28, 29). Their evolutionary dynamics are shaped by processes that differ from acute infections, including prolonged within-host adaptation, immune escape, recombination, glycan shielding, and latency or persistence (30–33). In such systems, the serological layer of a sites–structure–serology roadmap may need to be supplemented by within-host evolutionary models, cellular-immunity measurements, envelope glycan-shield analyses, reservoir or persistence dynamics, and transmission-bottleneck models. Thus, the sites–structure–serology triad should be viewed as a flexible template rather than a universal model: the relative weight assigned to each layer depends on the biology, epidemiology, and available measurements for the virus in question.

**Fig 1:**
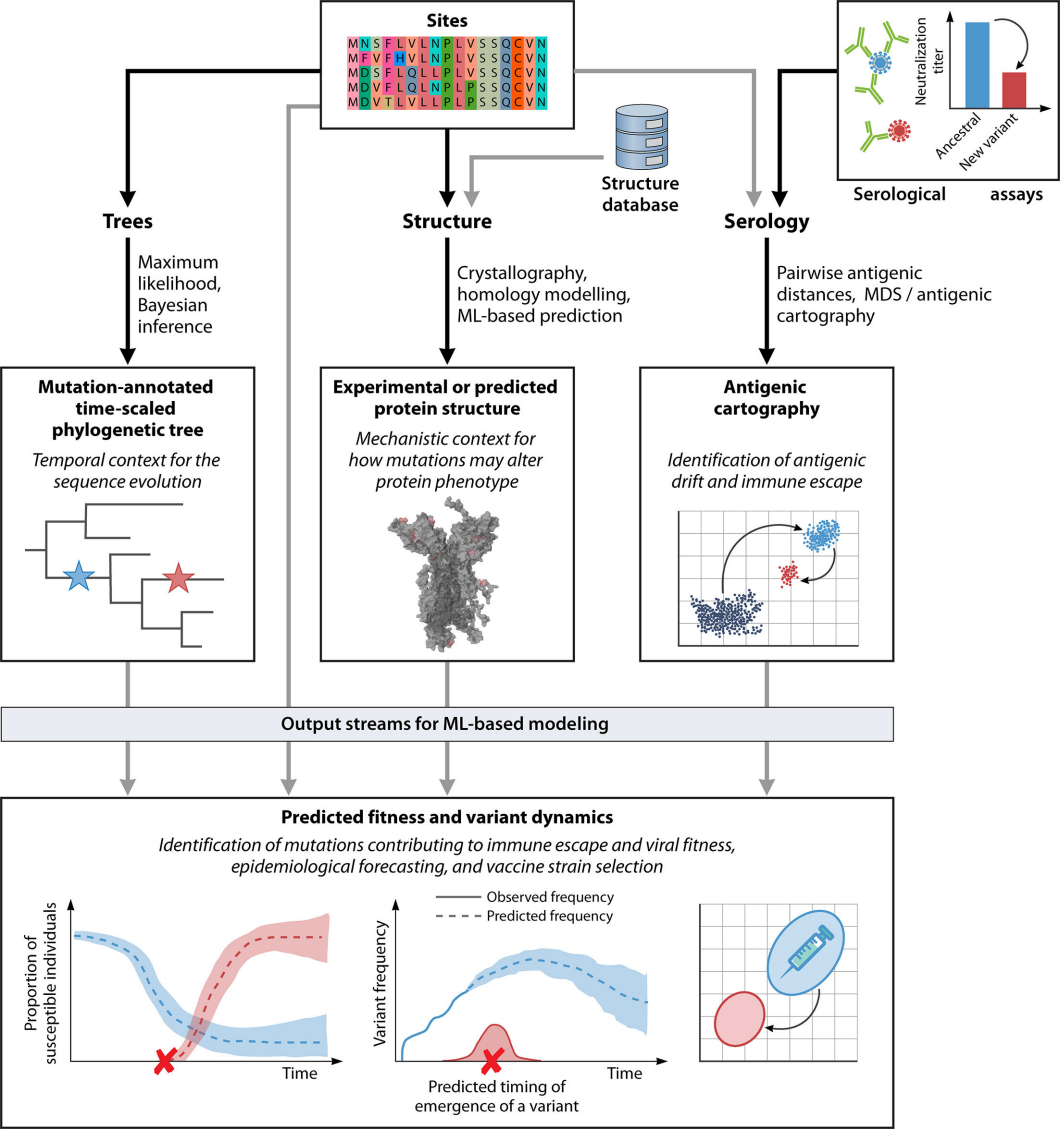
Integrative framework linking viral sequence variation to fitness and variant dynamics through machine-learning-based modeling. Schematic overview of how amino-acid mutations in viral genomes can be connected to predictions of viral fitness and population-level variant dynamics. Sequence data are used to reconstruct time-scaled phylogenies, providing temporal context for mutation emergence and lineage diversification. Mutations can also be mapped onto experimental or predicted protein structures to characterize features such as solvent accessibility, glycosylation sites, receptor-binding interfaces, and antibody epitopes. Serological measurements, typically organized as serum-virus titer matrices, can be converted into antigenic distances and visualized through antigenic cartography to quantify antigenic drift and immune escape. These phylogenetic, structural, and serological data streams can then be used independently or jointly as inputs to machine-learning models that estimate mutational fitness effects, forecast variant dynamics, and inform vaccine strain selection. Antigenic novelty can, in turn, be linked to population susceptibility through immune landscape models and related to lineage-frequency trajectories over time. The lower part of the schematic illustrates how antigenic predictions can be connected to population-level dynamics. Predicted antigenic novelty, together with information on the immune landscape of the host population, can be used to estimate changes in susceptibility to emerging variants. These susceptibility changes can then be related to observed or projected lineage-frequency trajectories, providing a bridge between molecular predictions, immune escape, and epidemiological dynamics. The arrows should be interpreted as a conceptual workflow rather than a single fully specified model; in practice, each link requires virus-specific data, assumptions, and validation. The schematic protein structure was obtained from the AlphaFold database (34, 35).

In the sections that follow, we first describe how large-scale genomic surveillance and time-resolved phylogenies quantify mutation supply and patterns of substitution. We then connect these patterns to fitness and antigenic evolution, introducing the distinction between genetic and antigenic clocks. Next, we discuss how protein structure helps explain unequal antigenic effects, pleiotropic trade-offs, and epistasis. Finally, we outline how machine learning (ML)—trained on sequences, serology, phylogenetic trees, and large protein sequence data sets among other data sources—can help bridge genotype, structure, and antigenicity, with an eye toward more prospective surveillance and effective vaccine and therapeutic design (Fig. 1). Key terms used throughout the review are defined in Table 1.

**TABLE 1:** Key terms used in structure-aware antigenic evolution

Term	Working definition
Antigenic distance	A measure of how differently two virus variants are recognized by a given serum panel. Antigenic distance is measured in “antigenic units” (AU), where 1 AU represents a twofold dilution in the laboratory assay. The metric is, therefore, generally dependent on the assay and serum panel.
Antigenic cartography	A framework for converting serological assay measurements, such as hemagglutinin inhibition or neutralization titers, into a low-dimensional map in which distances approximate antigenic distance between variants of the virus.
Antigenic drift	A model proposing that viral escape from population immunity occurs through the gradual accumulation of mutations in surface proteins.
Antigenic thrift	A model proposing that population immunity primarily targets epitopes with limited variability.
Immune landscape	The distribution of immune histories in a host population, shaped by infection, vaccination, waning, imprinting, and demographic structure.
Deep mutational scanning	A high-throughput experimental approach that measures the effects of many mutations independently, often across a whole virus protein, on phenotypes such as receptor or antibody binding, cell entry, or protein stability.
Variant effect predictor	A computational model that estimates the likely functional or fitness effect of mutations, often using evolutionary conservation, sequence context, structure, or experimental training data.
Protein language model	A machine-learning model trained on large protein sequence data sets to learn statistical representations of protein sequences that can be used for prediction tasks such as mutational effect or antigenicity prediction.
Pleiotropic trade-off	A situation in which a mutation (or genetic locus) has multiple phenotypic effects, such as increasing antibody escape while reducing receptor binding or protein stability.
Epistasis	Background dependence in mutational effects, where the effect of one mutation depends on the presence or absence of other mutations.
Structure-aware modeling	Modeling that incorporates three-dimensional or biophysical information, such as residue exposure, packing, stability, glycosylation, or binding interfaces, rather than relying only on sequence.

## FROM MUTATION SUPPLY TO PATTERNS OF GENETIC CHANGE

### Heterogeneous mutation and substitution processes

Viral genomes do not mutate in a uniform, site-independent way. Even in viruses with well-characterized genome-wide mutation rates, the probability that a given nucleotide mutates can vary substantially across sites (36). Part of this heterogeneity reflects local sequence context: neighboring bases modulate polymerase error patterns and the action of host editing enzymes such as apolipoprotein B mRNA editing enzyme (APOBEC) and adenosine deaminase acting on RNA (ADAR), giving rise to characteristic mutational spectra (37–39). In SARS-CoV-2, for example, C → U and G → U changes show strong motif dependence (39–41). Over longer timescales, genome composition is also shaped by selection, including depletion of CpG dinucleotides, which is thought to reflect selection against CpG-rich RNA and may involve zinc-finger antiviral protein (ZAP)-mediated antiviral pressure (42, 43). Rates of observed change also vary by genomic region, with structural proteins, non-structural proteins, and untranslated regions experiencing different functional constraints, RNA structure, and host factors (36). RNA secondary structure further shapes these patterns: base-paired positions and long-range interactions can be functionally constrained and differentially exposed to editing enzymes, affecting both the generation of mutations and their probability of being retained as substitutions (36, 44).

A related question is how this heterogeneous mutation supply translates into observed substitutions at different biological scales. For influenza viruses and SARS-CoV-2, most between-host evolution is driven by acute infections, in which infections are short-lived, transmission bottlenecks are tight, and within-host diversity is typically low and shaped largely by mutation and short-term purifying selection (45–48). In this regime, only a small and often stochastic subset of within-host variants contributes to the consensus-level phylogeny. By contrast, unusually long infections can accumulate extensive within-host diversity over many months, including convergent change at key sites (49, 50), which, in turn, may occasionally seed onward transmission of highly diverged lineages (51–53). Spillover and spillback events introduce additional shifts in mutational spectra and selection pressures when viruses replicate in new host environments (47, 54–56).

### From mutations to substitutions: selection on trees

Not every mutation that arises becomes an observable substitution. The probability that a mutation reaches appreciable frequency depends on its mutational supply and on the joint action of drift and selection (57, 58). Standard evolutionary summaries include the ratio of nonsynonymous to synonymous substitutions (dN/dS ratio) (globally, by gene, or site-wise) (59, 60), tests for recurrent or parallel change at the same codons in independent lineages (61, 62), and tree-based correlates of growth such as local branching metrics (63) or phylodynamic estimates (64, 65). Regions under positive selection typically show elevated nonsynonymous rates and recurrent changes, whereas core functional domains and many internal proteins often show strong purifying selection with comparatively few nonsynonymous substitutions (66–70).

Importantly, selection signals often localize to the same genomic regions that are epidemiologically salient. For influenza and SARS-CoV-2, concentrated nonsynonymous change in surface glycoproteins frequently overlaps known or putative antigenic regions. At this stage, however, such analyses tell us primarily where selection appears to be acting, rather than what phenotypic components of fitness are driving it. We return to the interpretation of these patterns—that is, what selection is acting *for—*in section *‘Fitness components and antigenic evolution’*.

### Molecular clocks and local rate heterogeneity

The molecular clock is the idea that substitutions accumulate approximately linearly with time along lineages, allowing genetic divergence to be converted into calendar time (71). In viruses, clock behavior can be highly heterogeneous. Even restricting attention to synonymous changes, substitution rates vary across sites and genes owing to differences in mutation rate, codon usage, RNA structure, and functional constraints (36–39). At the amino-acid level, a strong structural signal emerges: evolutionary rates tend to be lowest in the solvent-inaccessible, tightly packed protein interior, and highest at solvent-exposed surfaces where more alternative residues can be tolerated; functional constraints can locally override this trend around active sites and interfaces (72–74). These structural and functional gradients produce a landscape of local clocks, with some sites effectively frozen and others turning over quickly (67, 70, 75).

In viruses in which pre-existing immunity strongly shapes lineage turnover, this rate heterogeneity motivates comparing genetic change to phenotypic change in the trait most directly linked to susceptibility: antigenicity. We, therefore, distinguish two related but distinct “clocks”: (i) a genetic clock, measured as substitutions per site per unit time, and (ii) an antigenic clock, measured as movement in antigenic space per unit time (for example, in HI units on an antigenic map). The two clocks can correlate in epitope-rich regions but need not align (7, 76), because many genetic changes are antigenically silent and some antigenic shifts arise from a small number of structurally consequential substitutions.

**Fig 2:**
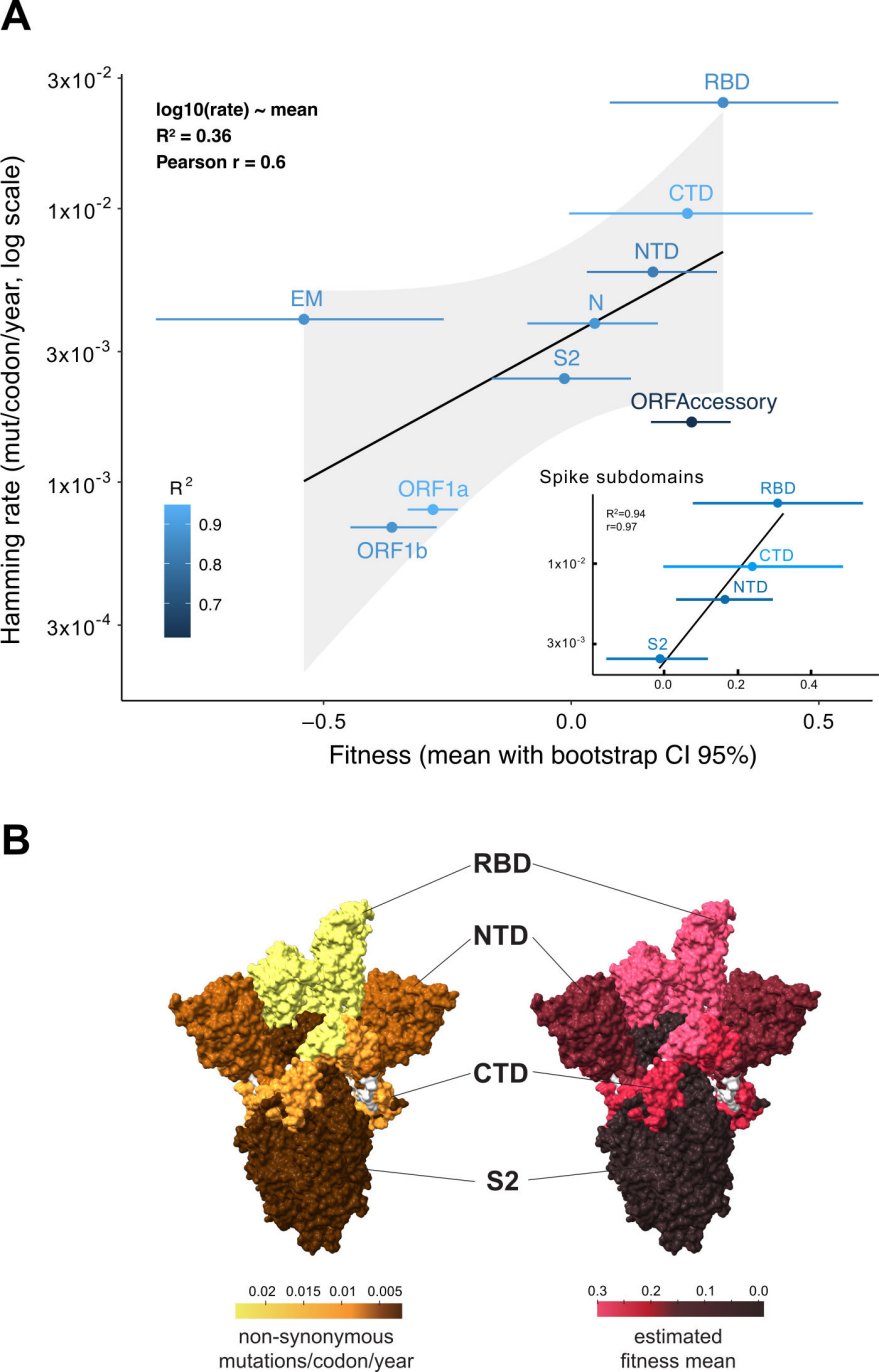
Regional nonsynonymous root-to-tip (Hamming) rates tend to increase with inferred mutational fitness effects, with the clearest relationship across spike subdomains. SARS-CoV-2 phylogeny and multiple sequence alignment were obtained from Nextstrain (85, 86) (downloaded 28 November 2025), where sequences had been preprocessed and aligned using Nextclade (87). After excluding non-human samples and clades lacking published fitness estimates, the final data set comprised *N* = 2,379 sequences sampled between 13 February 2020 and 26 March 2025. For each genomic region, nonsynonymous root-to-tip rates (mutations per codon per year) were estimated by regressing Hamming distance from the Wuhan-Hu-1 reference against sampling date, including only sequences with ≥90% coverage in the relevant region. Genomic partitions with root-to-tip regression fit *R*² >0.5 were retained as separate regions, provided that the 95% confidence interval of the corresponding fitness estimate was not larger than the overall range of mean fitness values across regions; otherwise, regions were merged. The final partitions were open reading frames 1a and 1b (ORF1a and ORF1b), RBD, N-terminal domain (NTD), C-terminal domains 1 and 2 (CTD), S2 gene, genes encoding envelope and membrane proteins (E + M), gene encoding nucleocapsid protein (N), and accessory open reading frames (accessory ORFs). Mean fitness effects of mutations were obtained from reference 88 (version 2024-11-06) and averaged within each genomic region, with 95% confidence intervals estimated by bootstrapping with replacement (1,000 replicates). (**A**) Across broad SARS-CoV-2 genomic regions, nonsynonymous root-to-tip rate is positively associated with the mean inferred fitness of mutations in each region. Horizontal bars show 95% bootstrap confidence intervals for the fitness summary; point color indicates the goodness-of-fit (*R*²) of the root-to-tip regression used to estimate the clock rate. The inset shows that the relationship is especially clear across spike subdomains (NTD, RBD, CTD, and S2), where regions with higher inferred fitness also show faster nonsynonymous turnover. (**B**) Spike structure colored by nonsynonymous root-to-tip rate (left) and mean inferred mutational fitness (right), illustrating the concordance between regional fitness and rate of genetic change within spike.

For molecular rate inference, multiple statistical frameworks are available. The key distinctions among commonly used approaches are how they account for phylogenetic uncertainty and whether they allow for rate heterogeneity among lineages (77–81). In the illustrative analysis of SARS-CoV-2 shown in Fig. 2, we use a simple descriptive metric—the root-to-tip (RTT) regression rate based on the absolute number of nonsynonymous mutations in each query sequence relative to the Wuhan-Hu-1 reference (MN908947.3)—to approximate the rate of genetic change over time across different genomic regions. Over short timescales of outbreaks with dense genomic sequencing, where relatively few mutations accumulate along each branch, a Hamming-based RTT summary can be used to measure divergence rate from the root (82). However, it should not be treated as a formal molecular dating approach. Where the goal is robust evolutionary rate inference, hypothesis testing, or comparison across more divergent genomic samples, it is preferable to use explicit substitution models and dating frameworks that account for phylogenetic structure, uncertainty, and rate variation among lineages (83, 84).

The approaches discussed in this section treat selection mainly as an implicit driver behind where and how rapidly substitutions accumulate, without yet specifying the phenotypic components of fitness on which it acts. To interpret those patterns mechanistically, we next focus on antigenicity as one of the tractable, epidemiologically relevant phenotypes that contribute to fitness.

## FITNESS COMPONENTS AND ANTIGENIC EVOLUTION

### Decomposing viral fitness

From a population-genetic perspective, fitness is a property of genotypes: it describes the expected contribution of a genotype to future generations relative to competitors. For pathogens, this genotype-level fitness is not observed directly; instead, it is inferred from changes in the frequency of mutations or variants, commonly summarized by a lineage-specific growth rate or effective reproduction number in the host population (89–91). While these operational measures act as proxies for underlying genotype fitness, they represent a composite outcome of multiple biological processes (such as cell entry, receptor usage, fusion, replication kinetics, tissue tropism, virion stability, shedding, transmission route, innate immune antagonism, and escape from adaptive immunity) rather than a single mechanistic trait. Many of these traits are governed by proteins that are not the dominant antibody targets, including polymerases, nucleocapsid proteins, accessory proteins, and non-structural proteins, as well as by genome-level features such as codon usage, CpG content, RNA secondary structure, and regulatory elements. The relative importance of these components varies across viruses and epidemiological contexts: immune escape may dominate lineage turnover in some immune-experienced respiratory virus populations, whereas receptor adaptation, replication competence, vector competence, latency, or within-host immune dynamics may dominate in other systems. We therefore focus on antigenicity because it is a measurable and epidemiologically important axis of fitness for the antigenically evolving viruses, not because it captures all determinants of viral success.

Biologically, the fitness of a genotype reflects the combined effects of multiple phenotypic components acting within a particular host and immune environment. Mutations can affect cell entry (receptor binding, cleavage, fusion), replication kinetics and tissue tropism (polymerase, nucleocapsid, and accessory genes), and transmission traits (stability and shedding dynamics), while immune escape modifies the effective susceptible pool by eroding protection from prior infection or vaccination (10, 21). These components are not independent: for example, escape mutations can impose stability or receptor-binding costs that require compensatory change (45, 92, 93). Moreover, the host immune landscape can strongly influence viral evolution, generating geographic variation and complicating efforts to model the spread and success of antibody-escape variants across regions and over time (75, 93, 94).

An illustrative consequence of this relationship is shown in Fig. 2. Broad genomic regions with higher mean inferred mutational fitness (88) tended to show faster nonsynonymous root-to-tip mutation accumulation rates (Fig. 2A). This relationship was clearest within spike subdomains, where regions such as the RBD combine repeated nonsynonymous turnover with strong adaptive selection, and as discussed below, antigenic evolution (Fig. 2B). By contrast, the weaker association outside spike—particularly in the shorter partition constituting envelope and membrane proteins (E + M) and the accessory open reading frames (ORFs)—likely reflects a mixture of stronger purifying selection and nonsynonymous changes that are tolerated, weakly selected, or highly context-dependent rather than consistently associated with lineage-level growth (88, 95, 96).

### Antibody escape as a key component of fitness

For endemic viruses circulating in immune-experienced populations, antibody escape is a central and tractable determinant of fitness. Serological assays such as HI for influenza and neutralization assays for SARS-CoV-2 quantify how well sera raised against earlier strains inhibit a new variant (11, 97). When summarized via antigenic cartography, these measurements yield an “antigenic distance” between viruses and an empirical record of antigenic drift over time (7, 11).

For viruses at or near a host jump, antigenicity is only one determinant of fitness, alongside traits such as host receptor usage, replication competence, and transmission potential. In contemporary H5N1 avian influenza virus, for example, sustained human-to-human spread is thought to require multiple adaptive changes beyond immune escape, including mammalian polymerase adaptation, altered HA receptor preference, and increased HA stability, and similar principles apply to zoonotic coronaviruses adapting to human transmission (98, 99). Even so, antigenic positioning relative to existing immunity and to candidate vaccine strains remains relevant in pre-pandemic contexts, and recent H5 antigenic cartography illustrates how explicitly mapping antigenic space can inform vaccine design (100).

A serum sample generally contains a mixture of antibody specificities targeting multiple epitopes, shaped by exposure history, age, and host species (101–103). As a result, antigenic maps and escape profiles derived from animal antisera may not perfectly match human serological landscapes, and different serum panels can emphasize different features of antigenic space (104). Recent high-throughput neutralization studies have begun to address this limitation by prioritizing human sera, thereby providing a denser and more representative view of the human antibody landscape (102).

Although antigenic cartography has been successfully applied to viruses for which neutralizing-antibody responses provide an interpretable measure of antigenic distance, such as influenza and SARS-CoV-2, its use is less straightforward across many other viruses. In some cases, antigenic relationships may depend on multiple proteins, conformational states, glycosylation patterns, serum exposure histories, or assay platforms, and measured titer reductions may not map directly onto protection. Other components of immunity, including cellular immunity, mucosal immunity, non-neutralizing antibody functions, or within-host immune escape, may therefore be more relevant than the antibody-mediated distances captured by standard antigenic maps. For example, in chronic infections caused by rapidly evolving viruses like HIV, antibody escape is shaped by extensive within-host viral diversification, glycan shielding, and complex host immune pressures, making simple population-level antigenic maps harder to interpret (105).

More broadly, antibody-mediated immunity represents only one component of protection. In viruses including influenza and SARS-CoV-2, T-cell responses can also contribute significantly to protection and shape evolutionary dynamics, but they are not directly captured by the serological frameworks that are the main focus of this review (106–108). In addition, even relatively modest antigenic differences may be associated with biologically meaningful differences in tissue tropism, replication site, or transmission potential (109). A further methodological limitation is that antigenic cartography relies on multidimensional scaling algorithms, which can introduce distortions in the estimation and representation of antigenic distances (110). Nevertheless, integrating antigenic cartography with controlled experimental challenge studies and other immunological measurements may therefore improve estimates of immune escape and vaccine performance (109). For respiratory viruses, where neutralizing antibodies remain a primary correlate of protection and vaccine strain updates are guided largely by serology, antigenicity provides a practical phenotype linking molecular change to population-level susceptibility. However, predicting antigenic evolution and antibody escape is only one component of vaccine effectiveness, which is also shaped by other factors, such as mucosal immunity, incomplete and variable correlates of protection, and the durability of immune responses to viruses (111).

### Genetic clock versus antigenic clock

**Fig 3:**
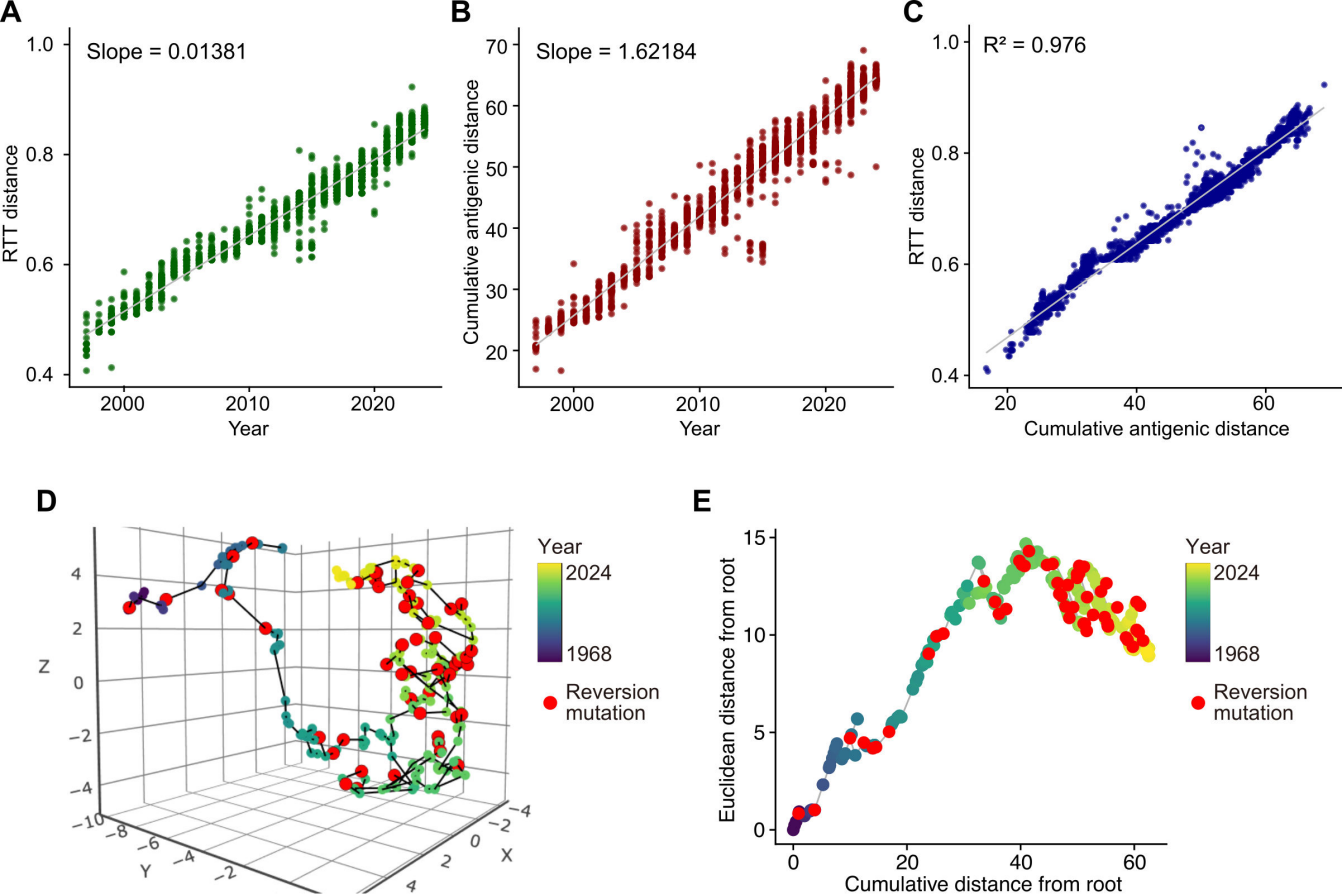
Genetic and antigenic trajectories along the evolutionary history of influenza A(H3N2). To characterize the genetic and antigenic patterns of influenza, HA sequences of H3N2 viruses together with corresponding HI assay data were obtained from Ito et al. (114). (**A**) Relationship between RTT genetic distance and year of virus sampling. Points represent external nodes (tips). Only virus samples collected after 1997 were included because of limited data availability for older strains. The slope was estimated by linear regression. (**B**) Relationship between cumulative antigenic distance and year of virus sampling. Cumulative antigenic change was calculated as the sum of antigenic changes along the branches comprising the phylogenetic trunk, defined as branches leading to internal nodes with ≥10 descendant tips, from the root to that node. (**C**) Relationship between RTT genetic distance and cumulative antigenic distance. (**D**) The phylogenetic trunk of H3N2 embedded in the PLANT antigenic map (114). Points represent internal nodes, lines represent phylogenetic branches, and point color indicates year. Internal nodes in which at least one amino acid reversion to the ancestral 1968 state occurred are highlighted in red. (**E**) Relationship between cumulative antigenic distance and Euclidean antigenic distance from the root during H3N2 evolution. All antigenic map coordinates, cumulative antigenic distances, and trunk-embedding analyses shown here are based on the PLANT analysis (114).

The genetic and antigenic clocks capture different summaries of change. Genetic divergence often increases roughly linearly with time along successful lineages, but antigenic divergence can be more irregular (Fig. 3). Empirically, antigenic and genetic distances are often correlated across long timescales (7, 112) (Fig. 3C), yet the relationship is not one-to-one: many substitutions are antigenically silent, while a small number of substitutions can produce large antigenic moves (7). Another mechanism that contributes to the decoupling of genetic vs antigenic clocks is reversion mutations: if antigenic phenotype is dominated by a small set of epitope residues, reversions at a subset of these sites can make later lineages appear antigenically closer to ancestral viruses even as overall genetic distance continues to increase (Fig. 3D and E; see also reference 113).

Seasonal influenza A(H3N2) is often described as exhibiting punctuated antigenic evolution: genetic divergence accumulates more continuously, but antigenic change frequently shows stretches of gradual drift punctuated by discrete cluster transitions. SARS-CoV-2 shows a somewhat different pattern. Major antigenic shifts have tended to coincide with episodes of unusually rapid genetic change during the emergence of new variants of concern (for example, Alpha and, more dramatically, Omicron), whereas within established clades, antigenic change can be comparatively modest even as substitutions continue to accumulate (10, 47). A key implication is that antigenic novelty is not determined simply by how many substitutions occur, but by which substitutions occur, where they lie in the protein’s structural and glycan context, and how they interact epistatically as part of a broader mutational constellation. The host environment can further shape the supply of such constellations. For instance, prolonged infections in certain individuals can permit extended within-host evolution under immune pressure in respiratory viruses (46, 49, 50) and, in the case of SARS-CoV-2, have been proposed as the main route by which highly diverged, immune-evasive lineages can arise and seed onward transmission (47, 51). However, the mechanisms by which intrahost selective pressures and immune responses shape population-level evolutionary and immunity patterns remain poorly characterized (115, 116). Integrating cross-disciplinary experimental assays that capture heterogeneous intrahost immune environments may help uncover mechanistic processes shaping viral evolution and epidemiological dynamics at the population level (115).

Taken together, these patterns suggest that sequence change alone is not enough to explain antigenic evolution. To understand why only some substitutions have large antigenic consequences, and why certain mutational constellations succeed where nearby alternatives fail, we need to consider the structural context in which those mutations act.

## STRUCTURE AS MEDIATOR: WHY ONLY SOME MUTATIONS MATTER ANTIGENICALLY

As discussed in the sections above, genomic features such as RNA secondary structure, long-range genomic interactions, packaging signals, untranslated regions, codon usage, CpG depletion, and exposure to host editing enzymes can all shape which mutations arise, which are tolerated, and which are retained as substitutions (117). These genome-level constraints may reinforce or conflict with protein-level requirements: a nucleotide change may be favorable at the amino-acid level but deleterious because it disrupts RNA structure, regulatory motifs, or genome packaging.

Protein structure also provides a key mechanistic context in which mutations act. Sequence-based analyses can identify where substitutions arise and how fast they accumulate over time, but they do not by themselves explain why some changes are antigenically consequential while others are tolerated, silent, or strongly deleterious. In viral surface proteins, the phenotypic effect of a substitution depends on its three-dimensional setting: whether it lies in or near an antibody-binding site, alters local surface geometry, changes glycan shielding, perturbs receptor binding, or destabilizes the fold (72, 74). Structure therefore helps resolve several of the central problems introduced above: why only a subset of genetic changes produce measurable antigenic effects, why mutational effects are often background-dependent, and why particular constellations succeed where nearby alternatives fail. In this section, we focus on three related questions: how mutations can be mapped onto 3D structures, how structural context shapes epistasis and trade-offs, and how combining structure with molecular evolutionary patterns can distinguish flexible escape routes from more constrained targets for vaccines and therapeutics.

Consequently, although sections *‘Structure as Mediator: Why Only Some Mutations Matter Antigenically’* and *‘Machine Learning at the Sites–Structure–Serology Interface’* primarily focus on protein structure, reflecting the central role of viral surface proteins in shaping antigenic phenotypes, a full structure-aware framework for molecular viral evolution ultimately requires integrating both protein biophysics and genome organization.

### Mapping mutations onto 3D structure

A practical starting point is to map observed or candidate amino acid substitutions onto experimental or predicted three-dimensional structures of the relevant viral proteins (e.g., influenza HA or SARS-CoV-2 spike). Each residue can then be annotated with structural and functional features such as solvent exposure, local packing, glycan occupancy, and proximity to known antibody footprints or functional interfaces, such as antibody-binding sites (10, 74, 118, 119).

Beyond annotation, structure is increasingly being treated as an evolutionary state space in its own right. A recent line of work converts predicted or experimental structures into discrete “structural sequences” (e.g., structural alphabets such as 3D interaction) and then fits substitution models directly in that representation, enabling likelihood-based structural phylogenetics with explicit structural substitution matrices (120–122).

### Constellations and epistasis in structural space

Structural context helps explain why some substitutions produce large antigenic effects while others are effectively silent. Mutations that directly alter antibody-contact residues, reshape local surface geometry, or change glycan shielding are more likely to shift antigenicity than substitutions that are buried or peripheral to dominant epitopes (119, 123, 124). In both influenza and SARS-CoV-2, these effects are constrained by pleiotropic trade-offs: mutations that improve immune escape can also reduce receptor binding, destabilize the fold, or perturb conformational dynamics, thereby limiting their evolutionary viability unless they are accompanied by compensatory changes (119, 124–127). In influenza, this is evident both within HA itself—where mutational effects at major antigenic sites depend on local receptor-binding constraints and genetic background—and between HA and NA, where epistatic interactions shape the evolutionary potential of antigenic change (124, 128, 129). In SARS-CoV-2, analogous compensation is seen in Omicron, where escape-associated RBD mutations that reduce ACE2 affinity can be rescued by interacting affinity-enhancing substitutions (125–127, 130).

More generally, novel variants carry constellations of mutations whose phenotypic effects are often epistatic rather than simply additive. At the structural level, such epistasis can arise through local packing interactions, long-range contacts, and shifts in conformational equilibria. Common motifs include escape-stability compensation, cooperative effects among residues within the same epitope, and allosteric or conformational epistasis, in which mutations shift the balance between structural states, thereby altering epitope presentation (23, 24, 131). A broader lesson is that even proteins with closely similar backbone architectures and shared biochemical functions can differ substantially in their site-specific evolutionary constraints and mutational effects, as illustrated by comparisons among HA subtypes (124, 132, 133). Taken together, these observations suggest that antigenic evolution is best understood not as the accumulation of independent site effects, but as movement through a structurally constrained and epistatic fitness landscape.

Predicted protein structures can now be used as a scalable “structural prior” for evaluating which mutational constellations are physically plausible (134, 135). In practice, one can predict structures for mutational combinations (or score their structural plausibility) and use resulting metrics—changes in predicted fold integrity, interface geometry, or stability proxies—to flag mutational combinations that are likely to be deleterious unless compensated (136, 137). Related approaches extend this logic from scoring mutations to designing and testing future antigenic trajectories. EVEscape integrates evolutionary and structural constraints to rank the escape potential of mutations and variants (138), whereas EVE-Vax uses a related framework to generate synthetic viral antigens that foreshadow future antibody escape and can be used to evaluate vaccine breadth prospectively (139).

### Conserved and immunogenic regions

Combining structural annotation with site-specific sequence conservation and evolutionary rate estimates per genomic region helps distinguish flexible “escape corridors”—surface regions that tolerate repeated change—from structurally and functionally constrained targets. This distinction is useful both mechanistically and practically: it can help explain why some regions of the antigenic surface repeatedly contribute to successful escape while others appear to be evolutionary dead ends, and it can help identify epitopes for broadly protective vaccines and therapeutic antibodies (140, 141).

A related implication is that antigenic trajectories need not be monotonic. Because antigenic maps are low-dimensional projections of a much higher-dimensional interaction landscape, and because antigenic phenotype can be dominated by a small subset of epitope residues, lineages can sometimes “loop back” toward an ancestral position in antigenic space via reversion mutations at key sites. One intuitive way to view this is that evolution is “confined” to a subset of viable antigenic phenotypes: early escape may move rapidly within an accessible region, but subsequent change is increasingly forced to explore along the edges of what is structurally and functionally tolerable.

Evidence supporting the loop-back hypothesis has been provided by a recent study by Ito and Kawakubo et al. (114). In that work, an antigenic map prediction model, termed PLANT, was developed using protein language models to infer antigenic map coordinates directly from H3N2 HA protein sequences, allowing reconstruction of an antigenic map spanning the full evolutionary history of H3N2. When the phylogenetic trunk of H3N2 was embedded into this antigenic map, the trajectory of antigenic evolution did not simply move linearly away from the root (Fig. 3D). Instead, after an initial increase in antigenic displacement, the path traced an arc around the root and the Euclidean distance from the root appeared to plateau even as cumulative antigenic change along the trunk continued to increase (Fig. 3E). Reversion mutations to ancestral amino acids were observed along this trajectory, suggesting that partial returns toward ancestral-like antigenic states at key positions may contribute to this pattern (58 reversion events in total; red dots in Fig. 3C and D). Taken together, these observations support the view that loop-back dynamics may occur during influenza antigenic evolution (113). More broadly, analogous non-monotonic antigenic behavior has also been suggested in other viruses such as dengue, where antigenically divergent lineages can periodically become more similar again, consistent with immune selection and functional constraints bounding evolutionary trajectories in antigenic space (142).

This kind of constrained, partially reversible exploration of phenotype space is reminiscent of the Prisoner of War model of molecular evolution (143), where the apparent accumulation of mutations can plateau or become non-linear when the observable evolutionary signal is bounded by constraint even while underlying change continues. In this analogy, the antigenic map is a 2D projection of a much higher-dimensional space, and the lineage is “imprisoned” within a cage defined by a combination of biophysical and immunological constraints: epitope geometry, glycan shielding, receptor-binding and stability constraints, and the host immune landscape itself, including immune imprinting, all of which restrict which escape routes remain viable (118, 144–146). Early on, movement within the cage can look directional; later, trajectories may slow as the lineages explore the boundaries of what remains structurally and immunologically accessible. Crucially, however, the cage itself may not be fixed. As population immunity changes through infection, vaccination, and waning, the fitness landscape can shift, effectively moving and reshaping the bounds of accessible antigenic space over time (147).

With this mechanistic view in place, we next consider how machine learning is increasingly used to link sequences, structures, and serological phenotypes at scale.

## MACHINE LEARNING AT THE SITES–STRUCTURE–SEROLOGY INTERFACE

### From genotype to antigenicity

A growing range of computational approaches are being used to link sequence variation to antigenic phenotype at scale. Broadly, these methods aim either to identify antigenically important mutations—for example, by estimating escape potential—or to predict antigenic relationships between strains (114, 148–151). More recent studies have introduced ML models that predict antigenic distances or related assay outcomes directly from sequence, in some cases using only information available from past seasons, for prospective evaluation (148, 149). Within this broader landscape, protein language model-based approaches (pLMs) represent a newer class of methods in which sequence embeddings can be used to predict escape mutations directly from viral protein sequence (150). Recent work has further used pLM embeddings to estimate mutation contributions and antigenic positions directly from sequence, thereby providing an explicit bridge between genotype and antigenic cartography (114).

An important strength of these approaches is that they can be embedded in explicitly temporal and epidemiological frameworks rather than being limited to static pairwise antigenic comparisons. In influenza, recent seasonal prediction frameworks show that sequence-based models can learn a genotype-to-antigenicity map from historical data alone and then use it prospectively to characterize newly circulating H3N2 variants (114, 148). In SARS-CoV-2, a new pLM approach combines sequence representations with deep mutational scanning (DMS) and spatiotemporal surveillance data to forecast mutation spread and variant dynamics at finer temporal and geographic resolution (152).

Despite these advantages, an important unresolved challenge is extrapolation under viral evolution. Because viruses evolve rapidly, newly emerging variants gradually move outside the sequence distribution represented in the training data, causing predictive performance to deteriorate over time. This has become an increasingly important evaluation strategy for genotype-to-phenotype prediction in viruses (148, 153, 154). In practice, iterative retraining can partly mitigate distribution shift, but it requires continued generation of new experimental data and frequent model updates. There is therefore a strong interest in models that are more robust to viral evolution and less dependent on continual retraining. More generally, these models also require careful evaluation: predictive accuracy depends on calibration to the underlying assay and serum panel, the extent to which animal sera measurements generalize to human immunity, validation strategies that respect both temporal and phylogenetic structure, and performance on genuinely out-of-distribution variants.

### Structure-aware ML for mutational effects

A major limitation of sequence-only models is that they do not explicitly represent the three-dimensional physical constraints under which viral proteins evolve. Yet, many of the phenotypes of interest, including immune escape, receptor binding, protein stability, conformational switching, and glycan shielding, are inherently structural. Structure-aware modeling seeks to address this by incorporating explicit geometric information to evaluate whether a mutation is structurally plausible and to suggest mechanistic reasons for its phenotypic effects (155–157).

Using experimentally determined structures from the Protein Data Bank (PDB) (RCSB.org) (158) and high-coverage predicted structures from resources such as the AlphaFold Protein Structure Database (AFDB) (https://alphafold.ebi.ac.uk/) (34), these methods combine sequence and geometric features or learn sequence–structure relationships directly. Hybrid models that explicitly integrate structural information to predict antigenic effects of mutations can improve interpretability by identifying conserved epitopes, estimating stability effects, modeling glycan gain or loss, highlighting candidate escape routes, and improving the interpretability of sequence-to-phenotype models (155–157).

At the same time, the effectiveness of current sequence-based models for viral mutation predictions remains under active evaluation (153, 154, 159). Recent studies have highlighted limitations in current models and training data, particularly for viruses, and have shown that performance may decline when applied outside their training domain or when benchmark design changes (153, 154). While fine-tuning with viral sequence data sets can improve model performance, the dependence of these models on site-level variation, and the relatively lower number of highly variable sites in viral proteins compared with cellular proteins, further constrain prediction accuracy (153, 160). Whereas so-called co-distillation frameworks, in which multiple pLMs learn from each other’s most confident predictions, may further improve the performance and generalizability of sequence-only variant effect predictors (VEPs) (161), structure-aware modeling remains an essential component of integrative prediction frameworks. By incorporating explicit geometric and biophysical constraints, structural information can improve the robustness and interpretability of predictions, particularly for phenotypes shaped by protein stability, receptor binding, glycosylation, conformational dynamics, and antibody recognition. However, structure should not be viewed as a stand-alone replacement for sequence-based, phylogenetic, serological, or experimental approaches; rather, it provides a mechanistic layer that helps connect sequence variation to phenotype.

### ML for fitness inference and forecasting

Beyond antigenicity, a growing class of models seeks to predict viral fitness and lineage growth more directly from sequence, structural, and contextual features. These approaches aim to prioritize mutations likely to spread and support early warning for high-risk variants. In this setting, fitness forecasting can integrate several complementary sources of information, including sequence-based predictors, alignment-based VEPs, structure-aware scoring frameworks, and experimental measurements from DMS (Table 2).

**TABLE 2:** Computational strategies for linking sequence, structure, serology, and fitness in viral evolution^*a*^

Information source	Main questions addressed	Representative classes of approaches	Strengths	Limitations
Sequence	Which mutations or sequence features are associated with immune escape or broader fitness relevance? Can genotype alone, or sequence-derived evolutionary constraint, predict antigenic relationships or candidate high-risk mutations?	Sequence-based antigenic predictors (148, 149); sequence-embedding and pLM-based antigenicity predictors (114, 150, 151); alignment-based evolutionary-constraint and variant-effect models (138, 162); broader biological sequence foundation models (163).	Highly scalable; sequence data are becoming more abundant and rapidly generated; suitable for prediction before full structural or serological characterization; can extract mutational fitness signals either from learned sequence representations or patterns and evolutionary constraints.	This is a methodologically heterogeneous category. Predictions can degrade for future variants outside the training distribution; sequence-only models do not explicitly encode 3D constraints, glycan shielding, receptor-vs-stability trade-offs, or immune history; risk of overfitting in small-N systems; current methodologies struggle to capture epistasis; performance of pLM-based predictors in sparsely sampled antigenic space remains uncertain; depend strongly on MSA depth and representativeness; foundation models are general-purpose and may require careful fine-tuning and calibration for virus-specific phenotypes; potential biosafety and biosecurity concerns arising from the dual use of biological data in model training (164).
Phylogeny	Where and when did mutations arise? How do substitution rates, lineage growth, and mutational fitness vary across sites, genes, clades, and over time?	Inference of mutational and clade-specific fitness effects (88, 165–167); clock rate variation (77–81); tree shape and clustering correlates of fitness effects (63, 168, 169); phylodynamic models (65, 170), with a growing number of machine-learning extensions for tree-based inferences (see Mo et al. and Braichenko et al. [171, 172]).	Adds temporal and evolutionary context; links mutations to lineage success; supports identification of genomic regions under adaptive or purifying selection; and enables explicit modeling of lineage turnover and rate variation. Some machine-learning approaches can also be faster and more scalable than likelihood-based methods once trained (171, 172).	Sensitive to sampling bias, recombination, phylogenetic uncertainty, and model misspecification. Machine-learning approaches may depend heavily on simulated training data, provide limited uncertainty quantification, and be sensitive to training assumptions or poor transfer across data sets (171, 172).
Protein structure	Which mutations are structurally plausible? Which sites are exposed, glycan-shielded, or functionally constrained? How do mutations affect protein stability, receptor binding, and antibody recognition?	Experimental and predicted structures (X-ray crystallography, cryo-electron microscopy, among other experimental methods [173], AlphaFold [135], ESMFold [134], and RoseTTAFold [174]); structure-aware Bayesian (157), structure-aware pLM (175), deep learning (155), and other hybrid (137, 156, 176) models.	Mechanistic interpretability, which is relevant to epitope geometry, glycan shielding, folding stability, and binding interfaces, helps filter implausible escape routes and identify conserved targets for vaccines and therapeutics	Structural data are sparse for many viruses; predicted structures may miss conformational ensembles and glycosylation details (121); training data bias (limited viral structures in PDB/AFDB); AlphaFold computationally expensive; ESMFold may trade speed for accuracy; more general intrinsic limitations due to low site-specific and between-site variation in viral data sets (153); methods potentially challenged by the higher-order epistasis; structure alone does not determine immune selection.
Serology	How antigenically distinct are different strains or lineages? How does genetic change translate into antibody escape and waning population immunity?	HI and neutralization assays; antigenic cartography (11); genotype-to-antigenicity models trained on titer data (114, 148, 157); genotype-to-antigenicity model trained on HI data for vaccine strain selection (177); high-throughput human-sera neutralization platforms (101).	Provides a directly relevant phenotype for vaccine updates and immune escape; connects sequence evolution to population susceptibility; identifies antigenically important mutations and variants.	Assays can differ across laboratories; measurements are noisy; human and animal sera may emphasize different regions of antigenic space; and serum panels reflect exposure history and immune imprinting, which can bias apparent antigenic relationships (178).
Experimental mutational assays	What are the functional effects of individual mutations? Which changes alter escape, receptor binding, expression, protein stability, or replication-related phenotypes?	Deep mutational scanning (179); predictive models trained on DMS (152, 180, 181).	Provides high-throughput functional ground truth; useful for training and validating predictive models; can identify trade-offs among escape, receptor binding, expression, and stability; offers a direct experimental route to mapping mutational landscapes (179, 182).	Often measures proxy phenotypes rather than organismal fitness; combinatorial mutation space (epistatic interactions) remains vast and so far largely unexplored; background dependence is incompletely sampled; currently available for only a subset of proteins and viruses; DMS assays may in general have limited value for fine-tuning pLMs because of their limited overlap with naturally circulating variants (183).
RNA secondary structure	Which nucleotide changes are constrained by RNA pairing, long-range interactions, regulatory motifs, packaging signals, codon usage, or dinucleotide composition? How do genome-level constraints interact with protein-level fitness effects?	Thermodynamic RNA-folding models; comparative RNA-structure prediction; SHAPE/DMS-MaPseq-guided models; deep-learning RNA secondary structure predictors (117).	Captures constraints invisible to amino-acid-level models; helps interpret synonymous and non-coding variation; relevant to site-specific substitution rates, codon usage, editing-enzyme exposure, and genome regulation (36).	Viral genomes can contain alternative, context-dependent, or long-range structures; predictions are sensitive to sequence length, pseudoknots, experimental conditions, and benchmarking strategy; integration with protein-level antigenic and fitness models remains underdeveloped.

^*a*^ Comparison of major information sources and modeling frameworks currently used to predict antigenic phenotype, mutational effects, lineage growth, and variant risk. For each class of approach, the table summarizes the primary information source, the questions it is best suited to address, representative method families and example tools, major strengths with potential application for real-time genomic surveillance and vaccine-update forecasting, and key limitations.

Alignment-based approaches are particularly relevant because they combine evolutionary constraint with structural and functional information to estimate escape potential and mutational fitness effects across diverse genetic backgrounds. EVEScape is a clear example of this logic, using prepandemic evolutionary information to forecast viral escape before a specific future variant has been observed (138). DMS-assisted approaches extend this by incorporating empirical measurements of mutational effects into predictive models of future spread. This is especially attractive for densely sampled viruses such as SARS-CoV-2, where large genomic surveillance data sets can be linked to functional measurements and epidemiological trajectories for a unified forecasting framework (152, 155, 180).

More broadly, these developments sit within a wider movement toward ML-assisted infectious disease forecasting (184). Existing approaches already span several model classes, including Bayesian genotype-to-antigenicity frameworks, sequence-based ML predictors, pLMs, alignment-based VEPs, and structure-aware scoring systems, each with different assumptions about what aspects of viral evolution are predictable (Table 2). A central challenge is to integrate heterogeneous evidence from genomic, structural, serological, and epidemiological sources, without losing interpretability. Just as importantly, these models must generalize to future variants that may occupy regions of antigenic space not represented in the training data. This is not only a methodological problem of out-of-distribution prediction, but also a biological question about how constrained or repeatable antigenic evolution actually is.

As in any machine learning application, small data challenges are also present in pathogen-related tasks, where they are often compounded by the limited availability of high-quality labeled data, incomplete or inconsistent metadata, strong regional and sampling biases, and the high dimensionality and evolutionary complexity of viral sequence data (185–187). These limitations can lead to overfitting and poor generalization, especially because viral evolution often involves non-additive interactions between mutations that conventional models struggle to capture.

Structure-aware and pLM-based models may help regularize this problem by encoding evolutionary or biophysical constraints, but their ability to predict non-additive effects in unsampled genetic backgrounds remains an open empirical question. Importantly, the success of large machine-learning models in other domains should not be assumed to translate directly to viral phenotype prediction. While pLMs can leverage abundant unlabeled sequence data to learn general evolutionary representations, their performance ultimately depends on the availability and quality of phenotypic data for calibration and evaluation. For many viruses, such data sets remain sparse and unevenly sampled, limiting confidence in predictions for unsampled regions of sequence space. In these settings, quantifying predictive uncertainty may be as important as improving predictive accuracy itself.

Current approaches to address these issues include transfer learning, self-supervised and semi-supervised learning, data augmentation, and combinations of deep learning with traditional machine-learning methods (185, 186). Future progress in viral phenotype prediction will likely depend on integrating complementary approaches that combine abundant sequence and structural information with limited phenotypic data, while incorporating biological and mechanistic constraints to better capture background-dependent and non-additive mutational effects. However, further progress will depend on improving model robustness, interpretability, and generalizability across understudied viruses, as well as on developing standardized, metadata-rich data sets that better integrate prior biological knowledge with sparse and heterogeneous viral data.

## OUTLOOK: TOWARD A STRUCTURE-AWARE EVOLUTIONARY SURVEILLANCE PIPELINE

An integrated framework for more accurate prediction of genetic and antigenic evolution of endemic viruses is now within reach. Dense sequencing and accurate reconstruction of viral phylogenies can identify emerging mutations; structural annotation can help interpret the biophysical and immunological constraints, escape corridors, and pleiotropic trade-offs that shape their viability; ML-based predictors can prioritize variants with possible antigenic or broader fitness consequences; and targeted experimental validation, including serology, DMS, and other functional assays, can be used to iteratively refine those models. Together, these approaches support earlier and more accurate identification and characterization of high-risk variants with implications for vaccine effectiveness and public health response.

At the same time, the current state of the field should not be overstated. Predictive frameworks, whether based on phylogenetics, structure, serology, experimental mutational data, ML, or combinations of these approaches, are still better at interpolation within already sampled sequence, structural, functional, or antigenic space than at genuine extrapolation to future variants. This is not only a technical limitation of current methods. It also reflects the stochastic nature of underlying molecular evolutionary processes in viruses: mutations are random and context-dependent, antigenic evolution is nonmonotonic, and shaped by shifting immune landscapes, so the predictability of future change is itself an empirical question. For this reason, the most valuable role of integrated predictive modeling may be to rank hypotheses, prioritize candidate high-risk variants for follow-up, and improve vaccine-strain selection, where even modest gains in vaccine effectiveness can translate into substantial reductions in disease burden (145).

Several methodological priorities for ML approaches follow from this. First, better training data remain essential. Although some newer models relax the need for deep multiple sequence alignments (MSAs) and can perform well on viral proteins (188), predictive performance still depends strongly on the quality, diversity, representativeness, and temporal relevance of the available training data. Recent virus-focused structure sources such as Viro3D (35) and BFVD (189) help address the longstanding underrepresentation of viral proteins in broader databases and should improve the structural foundation available for model development (122). Second, structure-aware approaches should be used more routinely, not because structure alone solves prediction, but because many of the phenotypes of interest, such as immune escape, receptor binding, protein stability, glycan shielding, and conformational change, are inherently constrained by the three-dimensional biology of the virus. Third, a better understanding and more systematic integration of protein–protein interactions, particularly virus–host interactions as well as interactions among viral proteins themselves, will be needed to move toward a more realistic prediction of evolutionary patterns and processes.

A practical bottleneck for implementing this roadmap is the absence of unified coordinate systems and interoperable data schemas. In many viral systems, connecting a mutation observed on a phylogeny to a codon in a reference genome, a residue in a mature protein, a position in a PDB or predicted structure, a glycosylation motif, an antibody footprint, a DMS measurement, and an antigenic-map coordinate requires substantial manual curation. Numbering schemes may differ between reference strains, alignments, mature proteins, subtypes, and structural models, and metadata standards for linking serology, structures, sequences, and epidemiological context remain underdeveloped. Building standardized, versioned, machine-readable coordinate maps should therefore be a priority for the next decade of evolutionary surveillance.

Other challenges still persist. Epistasis remains difficult to model in realistic genetic backgrounds, especially when only a small fraction of combinatorial sequence space can be sampled experimentally. Serological data remain sparse, noisy, and difficult to harmonize across host species and laboratories. For seasonal and vector-borne pathogens, environmental and climate-related drivers may also need to be incorporated more explicitly if forecasts are to achieve useful spatiotemporal resolution (184, 190, 191). More broadly, despite recent efforts to develop unified benchmarking frameworks that combine computational prediction models with experimental assay data and real-world viral sequence data (183), the field still needs standardized evaluation schemes that assess predictive accuracy, calibration, robustness to out-of-distribution variation, and mechanistic interpretability (153, 154, 192). We also need more systematic evaluation and a clearer understanding of the situations in which different model classes are genuinely useful, a challenge that is also present with the use of foundation models in biomedical research (193).

Finally, antigenic prediction should not be equated with vaccine efficacy prediction. Neutralization titers and antigenic distances are valuable correlates of immune escape, but protection against infection, disease, and transmission also depends on mucosal immunity, durability of immune responses, immune imprinting, T-cell immunity, non-neutralizing antibody functions, vaccine platform, dose, interval, and host population structure (194, 195). This is particularly important for respiratory viruses, for which durable sterilizing immunity is difficult to achieve (111). The framework we describe can therefore contribute to vaccine-strain selection and prioritization of variants for experimental testing, but it does not replace immunological, clinical, and epidemiological evaluation of vaccine performance. For the same reason, predictive models intended to support vaccine updates should ultimately be evaluated against the decision problem they are meant to improve. In seasonal influenza, this means assessing whether a model improves year-on-year retrospective vaccine-strain selection relative to appropriate non-ML baselines and to the antigenic and serological evidence already used in vaccine recommendation processes. This is especially important because contemporary A(H3N2) vaccine-strain selection relies heavily on experimental neutralization and serological data, including post-vaccination human sera, rather than sequence-based predictions alone (196, 197).

More broadly, many of the limitations discussed here are not unique to virology but may arise whenever ML approaches are applied to complex biological systems with limited labeled data and context-dependent genotype–phenotype relationships. Progress in viral evolution and antigenic prediction may therefore help guide the development of biologically informed predictive frameworks that integrate diverse sources of evidence while accounting for uncertainty. The larger goal, then, is an integrated evolutionary surveillance framework: one that connects sites, phylogeny, structure, serology, experimental assay data, and epidemiological context. Such a framework would not eliminate uncertainty, nor make virus evolution fully predictable. But it could make surveillance more hypothesis-driven, improve prioritization of emerging variants, and support more timely vaccine and therapeutic updates and public health decision-making.
